# ASSOCIATION AND RISK PREDICTION OF PAIN AND FALL-RELATED INJURIES AMONG COMMUNITY-DWELLING OLDER ADULTS IN CHINA

**DOI:** 10.1093/geroni/igad104.3188

**Published:** 2023-12-21

**Authors:** Xiaodong Chen, Kewei Shi, Lingxiao He, Ya Fang

**Affiliations:** Xiamen University, Xiamen, Fujian, China (People’s Republic); Xiamen University, Xiamen, Fujian, China (People’s Republic); Xiamen University, Xiamen, Fujian, China (People’s Republic); Xiamen University, Xiamen, Fujian, China (People’s Republic)

## Abstract

**Background:**

Pain is common in older adults and has also been identified as a fall risk factor, whereas the impact and mechanism of fall-related injuries are still unknown. This study aims to examine the association and risk prediction of pain and fall-related injuries among Chinese older adults.

**Method:**

This study enrolled 5,721 older adults from the China Health and Retirement Longitudinal Study (wave 2011-2015). Logistic regression was used to analyze the associations between pain characteristics and fall-related injuries. Furthermore, 2022 older adults with pain at baseline were further used to build a fall-related injury prediction model, using five machine learning algorithms (logistic regression, naive bayesian, random forest, adaptive boosting and artificial neural network) with 118 input variables as candidate features, and SHapley Additive exPlanations was used for the prediction model explanation.

**Results:**

Logistic regression models showed that whether pain (OR=1.35) or pain severity (ORmoderate=1.37, ORsevere =1.48) and multisite pain (OR=1.41) independently predicted fall-related injuries. The Random forest model achieved the best performance among the prediction model with an area under the receiver operating characteristic curve of 0.764. Interpretable results showed that the top 10 significant predictors were: experience of falling, frailty, sex, short physical performance battery, received inpatient care, estimated glomerular filtration rate, lung function, pain quantity, White Blood Cell and Age.

**Conclusions:**

Pain is associated with fall-related injuries among Chinese older adults. Fall-related injury prevention strategies for older adults with pain should specifically focus on fall history, frailty, and women.

